# The Effects of Whole-Body Photobiomodulation Light-Bed Therapy on Creatine Kinase and Salivary Interleukin-6 in a Sample of Trained Males: A Randomized, Crossover Study

**DOI:** 10.3389/fspor.2020.00048

**Published:** 2020-04-29

**Authors:** Jamie J. Ghigiarelli, Andras M. Fulop, Adam A. Burke, Anthony J. Ferrara, Katie M. Sell, Adam M. Gonzalez, Luke M. Pelton, Jamie A. Zimmerman, Shaquille G. Coke, Dennis G. Marshall

**Affiliations:** ^1^Department of Health Professions, Hofstra University, Hempstead, NY, United States; ^2^Crux Physical Therapy, Garden City, NY, United States

**Keywords:** recovery, low level laser therapy, heart rate variability, bed therapy, resistance training

## Abstract

Photobiomodulation therapy (PBMT) can be applied to the whole body as compared to the application of using single hand-held devices that isolate a smaller muscle area. The purpose of this study was to examine the effects of an acute dose of whole-body PBMT pre- and post-high-intensity resistance training on creatine kinase (CK) and salivary interleukin-6 (IL-6) in a sample of trained males. Twelve males (31 ± 8.3 years, 177.2 ± 5.4 cm, and 86.0 ± 7.5 kg) were part of a randomized, counterbalanced, cross-over design, whereby each participant performed a high-intensity resistance training session that consisted of the bench press, chin-up, and repeated sprints on two separate occasions. Each participant was assigned to either the PBMT or control condition on two separate weeks, with a 10-days washout period between the weeks. Creatine kinase was measured at baseline, 24, 48, and 72 h post-exercise. Salivary IL-6 was measured at baseline, 60, 90, and 120 min. A paired *t*-test showed no significant difference (*p* = 0.669) in the area under the curve (AUC) for CK during the PBMT (191.7 ± 48.3) and control conditions (200.2 ± 68.0). A Wilcoxon signed-rank test also showed no significant median difference (*p* = 0.155) in the AUC for salivary IL-6 during the PBMT (Mdn = 347.7) and control conditions (Mdn = 305.8). An additional Wilcoxon signed-rank test for CK percentage change from 24 to 72 h showed the PBMT condition (Mdn = −45%) to have a −18% median difference as compared to the control condition (Mdn = −41%). As such, whole-body PBMT does not significantly reduce the activity of salivary IL-6 or CK concentration during the 24 to 72-h recovery post-high-intensity resistance training.

## Introduction

Recovery from exercise is the act of re-establishing invested resources on a physiological and psychological level from periods of physical fatigue induced by training (Kellmann, 2002). The most common methods of recovery are cold-water immersion (CWI), contrast bath, stretching, nutrition, sleep, and active recovery (Crowther et al., 2017; Murray et al., 2017, 2018). A multidimensional approach is required, which entails monitoring several markers, such as cardiac parameters (i.e., morning heart rate variability), muscle damage biomarkers (i.e., creatine kinase [CK]), and hormones (i.e., cortisol) (Heidari et al., 2019). Photobiomodulation therapy (PBMT) has emerged as a potential method of recovery after training (Leal-Junior et al., 2019). Despite ambiguous and inconsistent terminology (i.e., “light therapy,” “cold laser,” LED, and near-infrared) in the research literature, in 2016, the term *photobiomodulation* was added to the database for Medical Subject headings in the National Library of Medicine. PBMT is defined as a non-thermal process used in therapeutic settings to alleviate pain, reduce inflammation, and promote tissue regeneration (Anders et al., 2015).

Systematic reviews in animal (Alves et al., 2014) and human models (Leal-Junior et al., 2015; Nampo et al., 2016; Machado et al., 2018; Vanin et al., 2018; Zein et al., 2018; Mussttaf et al., 2019; Wang and Wang, 2019) examined the efficacy of PBMT, which involves the application of light in the far-red to near-infrared spectrum (630–850 nm). Machado et al. (2018) and Vanin et al. (2018) report evidence of a possible beneficial relationship between PBMT on the management of CK and muscular performance (i.e., time to exhaustion, repetitions, isometric peak torque). In contrast, other reviews report PBMT has no beneficial effect on reducing muscle soreness (Nampo et al., 2016) and is not significantly different compared to placebo on muscular performance (i.e., repetitions, maximum voluntary contraction, peak force) and CK level (Wang and Wang, 2019).

PBMT is reported to reduce injury time (Foley et al., 2016), fatigue (Pinto et al., 2016; Lanferdini et al., 2018), cytokine activity (de Oliveira H. A. et al., 2018), and muscle damage (De Marchi et al., 2017) as well as improve sleep quality (Zhao et al., 2012). Not all of the research, however, is promising. PBMT is does not mitigate knee arthritis (Huang et al., 2015), and, recently, Vanin et al. (2018) highlighted inconsistencies in the methodology for clinical trials, including instrumentation of PBMT, wavelength dosing, heterogeneity within the population, and differences in exercise protocols. An additional concern is researchers are not consistent at reporting the PBMT device's technical parameters, including power density, energy density, and wavelength, as these parameters play an integral role the proper dosing of the therapy (Zein et al., 2018), and thus, the effectiveness of PBMT at the cellular level (Mussttaf et al., 2019). However, often times these parameters are not clearly reported in the scientific literature, limiting the generalizability, repeatability, and clinical interpretation of the results.

Studies that have examined the effects of PBMT on recovery and performance following exercise protocols include simple open-chain isolated single-joint exercises, such as knee extension (Antonialli et al., 2014; Baroni et al., 2015; de Paiva et al., 2016; De Marchi et al., 2017; Rossato et al., 2018) and elbow flexion (Larkin-Kaiser et al., 2015; Machado et al., 2017; Nausheen et al., 2017; Rigby and Hagan, 2019; Vieira et al., 2019). Studies have progressed to more dynamic protocols, such as such as in-game competitions (De Marchi et al., 2019; Dornelles et al., 2019), sport-specific tests (Pinto et al., 2016), running (Malta et al., 2016, 2018; Dellagrana et al., 2018; Peserico et al., 2019), plyometrics (Fritsch et al., 2016), and cycling (Teles et al., 2015; Malta et al., 2018). In addition, research has examined highly-trained sample populations, such as athletes in jiu-jitsu (Follmer et al., 2018), judo (Orssatto et al., 2019), volleyball (Ferraresi et al., 2015; da Cunha et al., 2019; Vieira et al., 2019), rugby (Pinto et al., 2016), water polo (Zagatto et al., 2016), and futsal (De Marchi et al., 2019). Much of the data support the ergogenic effect of PBMT, including reduced CK post-competition (De Marchi et al., 2019), improved time to exhaustion (Follmer et al., 2018), reduced muscle fatigue (Dornelles et al., 2019), improved rate of perceived exertion, and improved running economy (Dellagrana et al., 2018).

A review by Machado et al. (2018), concluded that PBMT does have a beneficial effect for the management of CK concentration when compared to control group findings. A critical finding is that PBMT is not effective at reducing CK concentration in general exercise (i.e., whole body) as compared to localized exercise. Other markers of acute physiological stress response to resistance training are used to implement recovery strategies (Jackman et al., 2018), in particular, a salivary marker interleukin-6 (IL-6). IL-6 is identified as the first “myokine,” that is in skeletal muscle and released from muscle contraction (Febbraio and Pedersen, 2005). Similar to CK, much of the literature documents that PBMT attenuates the activity of IL-6. In rat models, pre-exercise PBMT decreased IL-6 after aerobic training (Amadio et al., 2015) and resistance training (de Oliveira H. A. et al., 2018) and with experimentally induced knee inflammation (Pallotta et al., 2012). In a controlled trial, pre-exercise PBMT reduced IL-6 after eccentric knee extension in high-level soccer athletes (Vanin et al., 2016).

Most PBMT studies (92%) involved administering PBMT before exercise (Leal-Junior et al., 2015), with few studies that examined post-exercise therapy (Borsa et al., 2013; De Marchi et al., 2017; de Oliveira et al., 2017; Machado et al., 2017; da Costa Santos et al., 2019). Other studies have applied PBMT immediately before and after exercise (Antonialli et al., 2014; Miranda et al., 2018), and in recent studies, a combined treatment time is applied 6 h before and immediately before exercise (Rossato et al., 2018). Leal-Junior et al. (2019) recommend that PBMT therapy for acute effects from resistance training should be administered 5 min to 6 h before exercise and chronic effects, 5–10 min before each exercise session.

Typically, the conventional method of instrumentation is the use of a handheld device to treat localized muscle tissue (Teles et al., 2015; Pinto et al., 2016; Miranda et al., 2018; Vieira et al., 2019). As expected, larger devices, such as whole-body light beds (Zhao et al., 2012), have minimal research due to their cost (~$60,000–$130,000) but are assumed to be advantageous by treating more target tissue in a shorter amount of time. In addition, the administration does not require another practitioner or therapist to apply the treatment.

Currently, one study has applied PBMT using the whole body approach in a sample of women basketball players (Zhao et al., 2012). Zhao et al. (2012) reported PBMT increased serotonin secretion producing higher sleep quality scores and improved endurance performance compared to placebo group. Evidence is still lacking on examining high powered devices, using larger muscle groups, testing biochemical markers, and sampling athletic populations. Therefore, the purpose of this study was to examine the effects of pre-exercise and post-exercise whole-body PBMT on CK and salivary IL-6 in a sample of trained males. We hypothesized that using the whole-body PBMT therapy would result in reduced activity of CK and salivary IL-6 post-high-intensity resistance training compared to no PBMT.

## Materials and Methods

### Experimental Design

**Figure 1:**
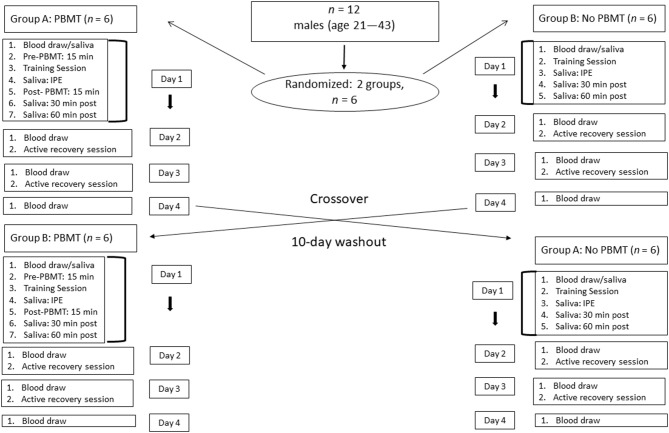
Experimental design flow chart.

A randomized, counterbalanced, crossover study was utilized to reduce treatment order bias (Figure 1). Twelve participants acted as their own controls and were randomized into one of two groups using the sequentially numbered sealed-envelope system (Vickers, 2006). Group A received PBMT in the first week, while Group B received PBMT in the second week. The area under the curve (AUC) was calculated to compare the differences in physiological responses of CK and salivary IL-6 during recovery between the PBMT and control conditions (void of PBMT). To determine the CK response, blood was collected at baseline, 24, 48, and 72 h post exercise. To determine the IL-6 response saliva was collected at baseline, 60 min (immediate post-exercise), 90 min, and 120 min.

These selected time points are similar to those of previous studies that examine the effect of PBMT on CK concentration (Felismino et al., 2014) and the responses of salivary IL-6 following strenuous exercise (Minetto et al., 2005, 2007; Usui et al., 2011). Due to high variability in saliva data, it is recommended that frequent sampling methods be performed, as this provides a closer relationship between saliva and serum concentrations (Papacosta and Nassis, 2011).

Similar to Miranda et al. (2016), a 10-days washout period was used to separate the experimental weeks. Participants performed their workouts at the same time of day on each of 3 days in both weeks. All blood draws and saliva samples were collected at the same time of day for both the PBMT and control weeks before and after the training session. Forty-eight hours prior to the high-intensity resistance training session, participants were instructed to refrain from any resistance training or aerobic exercise to ensure an accurate resting CK and salivary IL-6 level.

### Participants

Based on an a priori power analysis, using G^*^Power 3.1 software (Faul et al., 2007), we adopted a power of 0.80, α = 0.05, a correlation coefficient of 0.5, a non-sphericity correction of 1, and a strong effect (*f* = 0.4) of PBMT on CK and salivary IL-6 concentration. The rationale for the chosen effect size was based on priori power analyses from previous research using smaller 0.25 (Orssatto et al., 2019) and larger 0.52 effect sizes with a similar sample size to the current study (Peserico et al., 2019). From these values, a sample size of 12 was calculated using guidelines established by Beck (2013), which produced an actual power of 0.88 for the primary outcomes of CK and salivary IL-6. This sample size has been used in prior PBMT studies of a similar design using athletes (Pinto et al., 2016), and in studies that assess the pairwise comparison of two groups across multiple time points (four) during recovery on CK (Bartolomei et al., 2017).

Participants reported to the Crux Physical Therapy clinic (Garden City, NY) for a total of nine visits. Participant descriptives are depicted in Table 1. Prior to the first visit, participants were informed of all aspects of the study, obtained written consent to participate, and completed a written health screening to determine eligibility. Participants were recruited from a local training facility and university graduate sports science program. Participants came from varying sport backgrounds, consisting of CrossFit®, powerlifting, boxing, and cheerleading. Inclusion criteria were (1) male; (2) between the ages of 18 and 50 years; (3) having a minimum of 3 years of resistance training experience; and (4) free of any physical limitations, defined as having no upper or lower body musculoskeletal injuries that could affect the training sessions. Prior to enrollment, all participants completed the Physical Activity Readiness Questionnaire (PAR-Q) and a confidential medical and activity history questionnaire. Following an explanation of all procedures, risks, and benefits, each participant provided his informed consent form prior to participation in this study. Ethics approval was obtained from the local Human Research Ethics Committee 45 CFR 46.110(d) in accordance with the Declaration of Helsinki.

**Table 1:** Mean ± SD of participant's sport category, age, height, weight, one repetition maximum bench press (1RM BP), 1RM BP per body weight (BP/BW), and fat-free mass index (FFMI).

**ID**	**Category**	**Age (years)**	**Height (cm)**	**Weight (kg)**	**1RM BP (kg)**	**BP/BW**	**FFMI**
1	CrossFit	28	182.8	93.1	159.0	1.71	25.6
2	CrossFit	31	175.2	80.4	118.0	1.47	24.1
3	CrossFit	41	176.5	81.7	125.0	1.53	24.9
4	CrossFit	30	175.2	87.1	106.8	1.23	25.3
5	Powerlift	42	177.8	82.6	109.0	1.32	24.1
6	Boxer	21	175.2	85.4	113.0	1.32	22.9
7	Cheer	22	172.7	86.3	134.0	1.55	23.2
8	CrossFit	39	172.7	84.1	125.0	1.49	25.9
9	Powerlift	23	185.4	87.2	142.2	1.64	22.9
10	Powerlift	22	170.1	71.8	104.5	1.46	22.4
11	CrossFit	30	187.9	102.7	97.7	0.10	25.5
12	CrossFit	43	175.2	90.0	147.7	1.64	26.5
Mean ± SD		31.0 ± 8.3	177.2 ± 5.4	86.0 ± 7.5	122.6 ± 19.1	1.4 ± 0.2	24.5 ± 1.3

## Procedures

### Maximum Strength Testing

Prior to randomization, maximum strength testing was administered to obtain one repetition maximum (1RM) values for the barbell bench press. The 1RM testing protocol used is similar to that of previously published methods (Haff and Triplett, 2015). The 1RM value was used to properly load the participants for the bench press portion of the muscle-damaging protocol.

### Body Composition

Prior to starting the study, participants had their body composition tested through hydrostatic weighing (Get Tanked, Seattle, WA). Get Tanked is a mobile hydrostatic weighing clinic that provides scheduled appointments for individuals to obtain their body fat percentage, using a 300-gallon stainless steel tank. Body composition was tested to calculate the fat-free mass index (FFMI), which was used as a descriptor to verify the training status of the participants. The FFMI was calculated using a raw equation from Trexler et al. (2017). The tank is located in a Penske truck vehicle and has four hermetically sealed load sensors suspended from the floor of the tank. To obtain a reading, participants lie supine until their body is completely submerged for 7–10 s while breathing out as much air as possible. Once a body density reading is established, participants are signaled to come up for air. This trial is repeated 3–4 times. Hydrostatic weighing is a validated tool for body fat testing (van Marken Lichtenbelt et al., 2004).

### Muscle Damaging Protocol

During Day 1 of the PBMT and control weeks, participants underwent an exercise-induced muscle-damaging training session (Table 2). The training session consisted of the barbell bench press, chin-ups, and repeated sprints on a non-motorized treadmill (in this order). All participants were familiar with the selected exercises and the training intensities for the two training sessions. All participants were informed of the exercises for the muscle-damaging session and underwent a familiarization trial prior to the start of the study.

**Table 2:** Muscle-damaging protocol (Monday) and active recovery sessions (Tuesday and Wednesday).

**Day**	**Exercise**	**Resistance**	**Sets**	**Repetitions**		**Rest**
Monday	Bench press	70% 1RM	5	To failure		3 min
	Chin-ups	Bodyweight	5	To failure		3 min
	Repeated sprints	Bodyweight	4	4 6-s sprints; 30-s rest		144-s
		**Resistance**	**RPM**	**Sets**	**Work**	**Rest**
Tuesday	Cycling	None	50–60	14	1 min	1 min
Wednesday	Cycling	None	50–60	14	1 min	1 min

#### Bench Press

The bench press program was administered using previously published methods (Wilk et al., 2018). Before each training session, participants performed a general upper-body warmup with two sets of pushups and a horizontal abduction pull-apart exercise, using light resistance bands. Participants performed bench press specific warmup sets of 10 repetitions with the empty barbell, 5 repetitions with 40% 1RM, and 5 repetitions with 60% 1RM. For all training sessions, hand placement was self-selected and remained consistent in both training sessions. Participants were not allowed to bounce the barbell off the chest between the transition phase of eccentric and concentric contraction, and participants were instructed to keep five points of contact on the bench at all times. A metronome (KILQ Metro-pitch Digital Tuner Metronome, New York City, NY) was used to guide cadence and ensure proper tempo. Participants performed five sets of bench press, using 70% of their 1RM. Participants performed each set to failure with a 3-min rest in between sets. Participants were verbally encouraged throughout all training sessions to complete as many repetitions as possible.

#### Chin-ups

Participants performed the chin-up technique using previously published methods (Ronai and Scibek, 2014). The chin-up exercise was selected due to the multiple joints involved throughout the concentric and eccentric phases of the exercise, including the latissimus dorsi, serratus anterior, biceps brachii, brachialis, trapezius, rhomboids, pectoralis minor, posterior deltoid, and rotator cuff (Youdas et al., 2010). Participants started the exercise by grabbing the elevated bar on the rig apparatus with a shoulder-width supinated grip. Once participants obtained a firm grip and achieved a static hanging position, they pulled against their bodyweight until the underside of their chin was above the bar. After completing the ascending phase, participants slowly lowered their body back to the starting hanging position. Participants completed five sets of chin-ups to failure, with a 3-min rest in between sets. Participants performed the chin-up with a similar tempo as the bench press exercise, consisting of a 5-s eccentric phase followed by a 1-s concentric phase. A metronome (KILQ Metro-pitch Digital Tuner Metronome, New York City, NY) was used to guide cadence and ensure proper tempo.

#### Repeated Sprints

The procedure for the repeated sprint protocol was taken from Minahan et al. (2018), which is shown to increase CK levels 24 h post exercise. One week prior to the repeated-sprint sessions, participants were familiarized with the non-motorized treadmill. The familiarization involved 5 min of self-selected submaximal running on a non-motorized treadmill (Trueform Rogue Fitness, Columbus, OH), followed by five sprints (not maximum effort) for a 6-s effort with a 1-min rest intervals between the sprints. Participants were allowed as much time as necessary to remain on the treadmill once they were comfortable with sprinting from a stationary stance and straddling the treadmill after the 6-s sprint effort was completed. The repeated sprint protocol consisted of four sets of four maximal intensity 6-s sprints, with 30 s of rest between each sprint. After the fourth sprint of the set, participants were given a 144-s rest until the next set of four sprints. After the 6-s of maximal sprinting, participants safely straddled the treadmill by placing their hands on the side rails, thus avoiding the moving belt as it decelerated.

#### Active Recovery Sessions

During days 2 and 3 of the training week, participants performed a light active recovery session of ~30 min on the Assault Air Dyne Bike (Rogue Fitness, Columbus, OH). The recovery session consisted of 1 min of work, alternating with 1 min of rest (one set) for seven sets, followed by 5 min of passive recovery, then a repetition of seven sets. Participants were instructed to not engage in any other type of physical activity or recovery modality during the two recovery days.

### Whole-body Photobiomodulation

**Figure 2:**
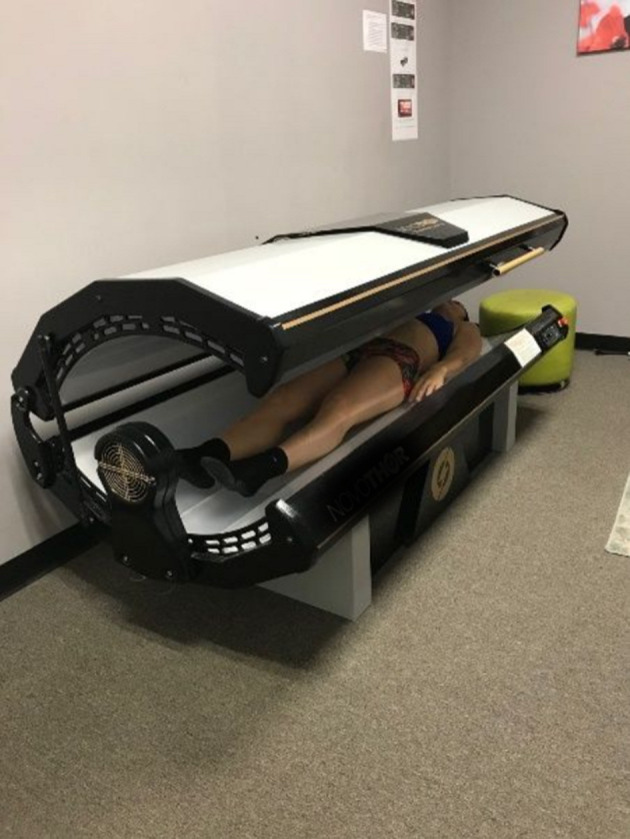
Pictorial representation of the photobiomodulation whole-body light-therapy bed.

The photobiomodulation sessions were administered using a whole-body PBMT bed (Figure 2, NovoTHOR® Hampstead, MD) at the Crux Physical Therapy clinic, which is located inside the local training facility where the training sessions took place. Participants suggested the use of a small fan, primarily for comfort, as it was getting uncomfortably hot in the treatment room. Therefore, a small fan, set on a low setting, was added. The technical parameters for the PBMT are listed in Table 3. The NovoTHOR is a whole-body LED bed that emits red (660 nm) and near-infrared (850 nm) light. The dimensions are 1.96 meters (m) length, 0.89 m width, and 1.32 m in height. Participants were provided with safety glasses and instructed on the use of the device. All parameters were set by the technician and initiated via a remote system. Generally, the manufacturer's treatment times in the NovoTHOR range from 8 to 20 min. Immediately before and after the muscle-damaging protocol, participants were instructed to lie supine in the bed for 15 min for each PBMT session with minimal attire (i.e., underwear). This timing for the moment of irradiation is in accordance with the guidelines of Leal-Junior et al. (2019) regarding clinical recommendations for the use of PBMT for exercise performance. During the control trial, participants laid supine in the bed with no light therapy for the same allotted time.

**Table 3:** Technical parameters of PBMT.

**Parameters**	**Specifications**
Number of LEDs	2,800
Wavelength	mixed, of 660 and 850 nm
Power output	2,700 W
Total power emitted	526 W
Treatment time	15 min
Beam area per LED (at the lens/skin contact)	6.5 cm^2^
Energy emitted per LED	164 J
Total energy emitted over 15 min period	473, 400 J
Power density (irradiance)	17 mW/cm^2^
Energy density (fluence)	25 J/cm^2^

### Blood Collection

**Figure 3:**
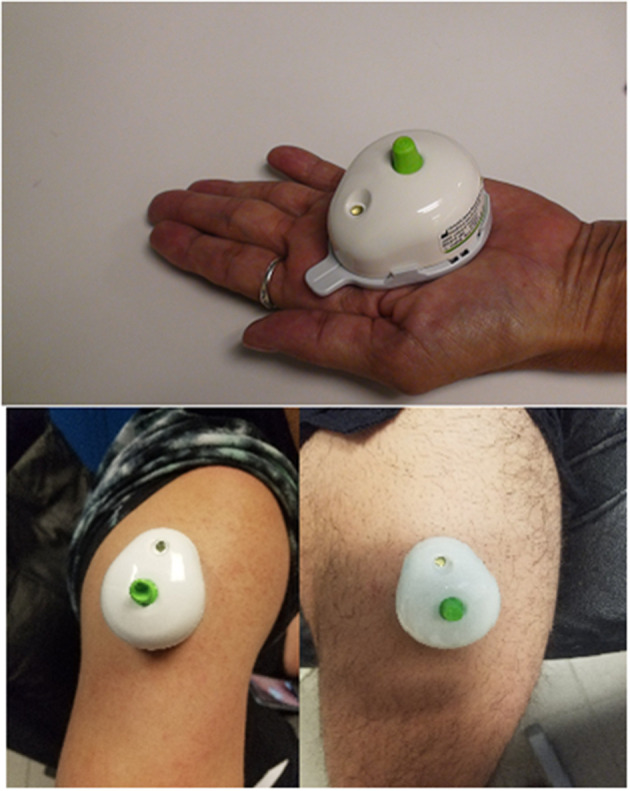
Pictorial representation of the Touch Activated Phlebotomy device in the upper arm and upper leg.

On days 1 through 4 of the training week, blood draws were performed, using a touch-activated phlebotomy device (TAP; Seventh Sense Biosystems, Medford, MA). The push-button blood collection device (Figure 3) was utilized to increase participant compliance and to ease the blood-collection process. The TAP is a lithium heparin-coated, single-use device intended to be used to collect capillary blood from the upper arm and upper leg. The device uses a combination of capillary action and vacuum extraction to obtain capillary blood samples for *in vitro* diagnostic testing. Capillary testing is a validated (de Oliveira D. C. X. et al., 2018) and accurate method to test for CK (Knoblauch et al., 2010). In addition, CK is a validated (Lee et al., 2017), easy-to-measure, cost-effective marker used to assess muscle damage or trauma from high-intensity exercise (Koch et al., 2014). The device allows for 100 μL of whole blood and uses microneedles to create skin-puncture access. The blood is collected in a reservoir, and an indicator confirms that collection is complete. The TAP device was approved by the Federal Drug Administration in 2017 for HbAlc, a biomarker to screen, diagnose, and monitor diabetes.

### Creatine Kinase Analysis

Two (upper arm and upper leg) TAP devices were placed in biohazard specimen bags to be refrigerated for 24–48 h at 0–5°C. Subsequently, TAPs were placed in 7 × 6 insulated containers with 3-oz. cold packs and 1-day shipped (United Parcel Service) to Seventh Sense Biosystems for sample processing. Upon arrival at Seventh Sense Biosystems, two TAP devices from the same donor were removed from the devices and pooled together into a single sample. The pooled samples were centrifuged at 3,000 rpm for 10 min to separate the plasma from the cells. After centrifugation, the plasma was carefully transferred by pipette into a separate microtube. Equal amounts of plasma and 0.9% NaCl (by volume) were mixed to create a 2× diluted sample with adequate volume for experimental testing. On the same day, the centrifuged samples were transported to Boston Clinical Laboratories (Waltham, MA) for CK analysis. Boston Clinical Laboratories is a full-service referenced laboratory that provides diagnostic testing to hospitals, physicians, and medical centers and is licensed by state and federal agencies by the College of American Pathologists to perform testing in clinical chemistry. The methodology used for the CK samples was enzymatic catalysis measurement with a low coefficient of variation of 0.5%.

### Saliva Collection and Analysis

Participants rested for 10 min before providing resting saliva samples. A second saliva sample was collected immediately post-exercise (60 min). The participants then relaxed for 30 min before supplying a third saliva sample (90 min post-baseline), followed by a fourth sample, 30 min later (120 min post-baseline). Participants were instructed to avoid hard foods, caffeine, amino acids, hot fluids, and brushing their teeth for ~60–90 min and were allowed to drink only water before testing to minimize any risk of contamination in the saliva. Participants replicated this sampling procedure for both training-day sessions.

Whole saliva samples were collected with SalivaBio's 2 mL cryovials and the Saliva Collection Aid (Salimetrics, San Diego, CA), a collection device specifically designed to improve volume collection and increase participant compliance. After 2 min, the primary investigator removed the passive drool tube from the 2 mL cryovial and then placed a cap on the tube to prepare for storage.

Samples were immediately stored at −20°C (CMF151L-EdgeStar) and later shipped to Salimetrics Inc. Samples were assayed for the salivary IL-6 in duplicate at the Salimetrics SalivaLab (Carlsbad, CA) using a proprietary electrochemilluminesence method developed and validated for saliva by Salimetrics. The average coefficient of variation for all samples tested was <15%, which meets the SalivaLab's criteria for accuracy and repeatability in Salivary Bioscience, and exceeds the applicable National Institutes of Health (NIH) guidelines for Enhancing Reproducibility through Rigor and Transparency. Sample test volume was 25 μL of saliva per determination. The assay has a lower limit of sensitivity of 0.0491 pg/mL for IL-6 with a dynamic range from 0.0491 to 736 pg/mL. Prior to testing, samples were stored at −80°C before being shipped on dry ice to the Salimetrics SalivaLab.

### Dietary Analysis

Participants were advised to maintain their normal diet and to record as accurately as possible everything that they consumed during the PBMT and control weeks. For the experimental and control weeks, participants were required to duplicate the content, quantity, and timing of their daily diet during each of the training days and active-recovery days. Participants were instructed to avoid alcohol and any supplements (i.e., creatine, branched-chain amino acids, and pre-workout drinks) during the study. Participants were instructed on how to properly complete a 3-days dietary recall log to include all food items and their respective portion sizes consumed. Dietary analysis software (MyFitnessPal^®^) was used to analyze dietary recalls, which was analyzed by a certified sports nutritionist (CISSN).

### Sleep Analysis

Participants were instructed to maintain their regular sleep pattern throughout the study period. All participants were instructed to wear the wrist-activity monitor (Readiband™; Fatigue Science, Vancouver, BC Canada) to record sleep time and sleep efficiency for the duration of the study, including the 10-days washout period. The Readiband™ is a Federal Drug Administration-approved (Dunican et al., 2018) actigraphy monitor that has been shown to accurately detect sleep/wake episodes in comparison with polysomnography, the gold standard of actigraphy measures (Dunican et al., 2017). It contains an accelerometer that tracks the frequency of wrist movements and, using a proprietary algorithm, processes these movements to measure and quantify sleep and awake periods and to measure the quality of sleep periods (McCormick et al., 2012). The Readiband Sync™ software was used to synchronize the data from the device. The primary investigator monitored all participants remotely and exported data for analysis.

### Morning Heart Rate Variability

Participants recorded their daily morning heart rate variability (HRV) for the duration of the study. HRV can be used to assess readiness status and monitoring of wellness and training adaptations (Williams et al., 2018). In addition, morning HRV can explain up to 25% of CK change (Weippert et al., 2018). Participants were instructed to perform a 120-s morning readiness HRV measurement each morning upon waking, in a seated position. The root mean square of successive R-R intervals (RMSSD) was used as the HRV measurement. A Polar H10 Bluetooth heart rate strap (Polar Electro, Kempele, Finland) was paired with an available smartphone application (Elite HRV, Ashville, NC) for daily HRV measurements. The Elite HRV application is a validated (Perrotta et al., 2017; Williams et al., 2018) and reliable (Perrotta et al., 2017) tool for measuring HRV. The primary investigator monitored all participants remotely and exported data for analysis.

### Statistical Analysis

Descriptive data for participant characteristics and experimental variables were calculated as means and standard deviations. A Shapiro-Wilks test was used to test for normality on all dependent variables. Paired *t*-tests were used to examine the differences in baseline scores to assess baseline CK and IL-6 values.

For CK and salivary IL-6 scores, AUC was calculated using GraphPad Prism version 8.2 for Windows; GraphPad software, La Jolla, CA. Paired *t*-tests were used to assess differences between AUC and percentage change for specific time points across both conditions. A non-parametric Wilcoxon signed-rank test was used when the data violated normality assumptions. Paired *t*-tests were used to examine the mean differences across the three training days for dietary analysis, morning HRV scores, and sleep scores for both conditions (Table 4) (Supplementary Tables 3, 4), along with the comparison between AUC CK levels across the first and second weeks. Data analysis was performed using IBM SPSS, Version 25.0 (SPSS Inc., Chicago, IL) software for Windows and statistical significance was set at α = 0.05.

**Table 4:** Mean ± SD for calories, carbohydrates, fats, and proteins; sleep time; sleep efficiency; and morning heart rate variability between the groups.

**Variable**	**PBMT**	**Control**	***p***
Calories	2462.5 ± 757.7	2523.8 ± 636.5	0.574
Carbohydrates (g)	224.2 ± 106.1	235.0 ± 74.3	0.591
Fats (g)	104.8 ± 26.4	106.3 ± 27.9	0.536
Proteins (g)	153.3 ± 67.7	157.8 ± 65.5	0.465
Effectiveness score (au)	88.5 ± 6.9	89.6 ± 5.3	0.371
Sleep efficiency (%)	86.3 ± 8.0	85.8 ± 7.69	0.784
lnRMSSD (ms)	4.1 ± 0.6	4.0 ± 0.59	0.525

*au, arbitrary units*.

## Results

### Baseline Characteristics

**Figure 4:**
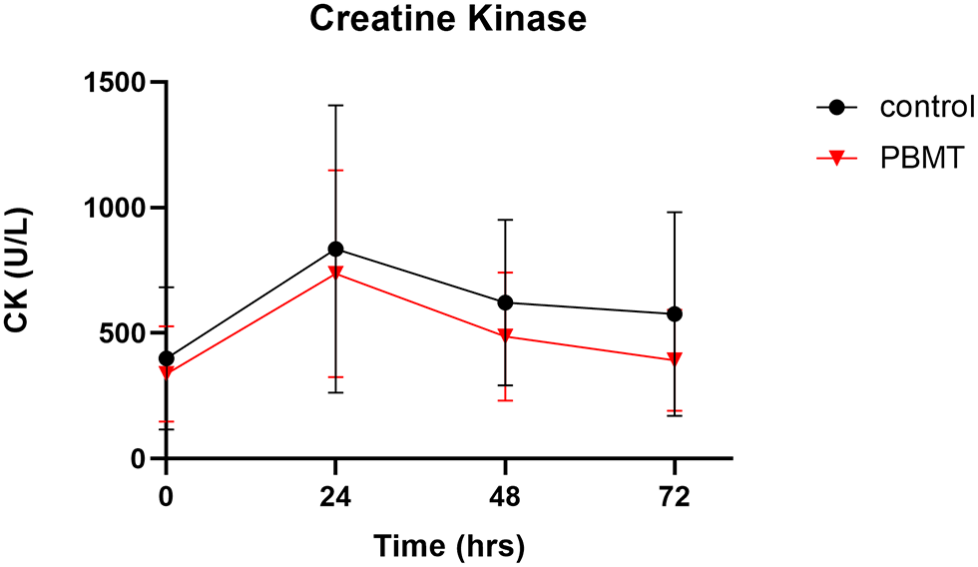
Creatine kinase concentration across baseline, 24, 48, and 72 h.

**Figure 5:**
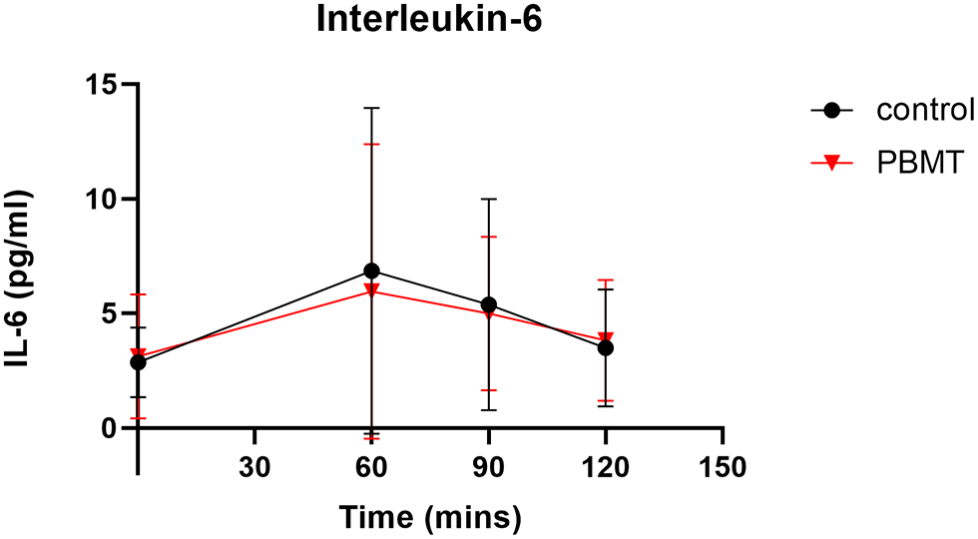
Interleukin-6 concentration across baseline, 60 (immediate post-exercise), 90, and 120 min.

There was no statistical difference in baseline CK values across the PBMT and control weeks (Figure 4) (Supplementary Table 1). Baseline CK values for the PBMT and control weeks were 341.0 ± 188.5 U/L and 387.0 ± 292.2 U/L, respectively (*df* = 11, *t* = 0.526, *p* = 0.585, mean difference = −46 ± 283.3 U/L, 95% CI:−134.0–226.0). In addition, there was no statistical difference in baseline IL-6 values across the 2 weeks (Figure 5) (Supplementary Table 2). Baseline salivary IL-6 values for the PBMT and control weeks were 3.11 ± 2.7 pg/ml and 2.85 ± 1.52 pg/ml, respectively (*df* = 10, *t* = 0.427, *p* = 0.678, mean difference = 0.259 ± 2.0 pg/ml, 95% CI: −1.09–1.61).

### Creatine Kinase

There were no outliers, as assessed by the examination of studentized residuals for values >± 3 standard deviations. The AUC was calculated across the four time points for both conditions. The AUC for PBMT violated normality, as assessed by a Shapiro-Wilks test (*p* = 0.038), due to positive skewness; therefore, a square-root transformation of the data was applied to both scores to meet the normal distribution assumption. After data transformation, a paired *t*-test showed that the square root AUC for PBMT (191.7 ± 48.3) was not statistically different (*df* = 11, *t* = 0.440, *p* = 0.669, mean difference = −8.3 ± 66.1, 95% CI: −50.4–33.6) from that of the control (200.2 ± 68.0).

Percent change calculations from 24 to 72 h showed that, of the 12 participants in the study, seven participants had greater decreases in CK on the PBMT occasion than on the control occasion, whereas five participants had greater decreases in CK on the control occasion. Due to a violation of normality for the percent change score for PBMT (*p* = 0.006), a non-parametric Wilcoxon signed-rank test was used to determine that there was no significant difference in percent change for CK (*df* = 11, *z* = −1.80, *p* = 0.071, effect size [r = (z/√n) = −0.38] when participants used the PBMT treatment compared to control. The median of the differences was −18% when participants used the PBMT treatment compared to the control. The median decrease was −45 and −41% for the PBMT and control condition, respectively.

The AUC for the second week was not statistically significantly lower than that of the first week, albeit there was a moderate effect size (d = 0.54); first week (47991.0 ± 26088.1), second week (34984.7 ± 20944.1), *df* = 11, *t* = 1.874, *p* = 0.08, mean difference = 13006.2 ± 24039.4, 95% CI: −2267.6–28280.1.

### Interleukin-6

There was one participant with outlier values, indicated by studentized residuals >± 3 standard deviations; thus, this participant was removed from the analysis. The AUC was calculated across the four time points for both the PBMT (345.2 ± 274.2) and control (381.4 ± 316.8) groups. The AUC scores for PBMT and control conditions violated normality (*p* = 0.001 and *p* < 0.0001, respectively). A non-parametric Wilcoxon signed-rank test showed that the AUC for salivary IL-6 was lower (*df* = 10, *z* = −1.42, *p* = 0.155) in the control group (Mdn = 305.8) as compared to the PBMT group (Mdn = 347.7).

## Discussion

This study aimed to examine the recovery response of CK and salivary IL-6 from an acute dose of whole-body PBMT light-bed therapy administered before and after high-intensity resistance training in a sample of trained males. The significant findings were that PBMT did not significantly reduce CK or salivary IL-6 response post-exercise compared to the control group; thus, our primary hypothesis was rejected. Despite the lack of a significant difference between the conditions, a notable finding was the median of the difference score was −18% for CK change from 24 to 72 h when the PBMT was compared to the control condition, producing a moderate effect size.

The underlying theory of the ergogenic mechanisms of PBMT is that far-red and near-infrared photon energy is absorbed by the subcellular chromophores of the mitochondria. This triggers the enzyme cytochrome c oxidase, which accelerates ATP synthesis in the mitochondrial respiratory chain (Huang et al., 2009). This accelerated ATP synthesis subsequently increases the proliferation of myoregulatory factors, such as myogenin and MRF4, which are integral to the formation of mature muscle fibers and muscle repair process (Alves et al., 2014). For athletes, the “muscle protection” effects of PBMT involve reducing inflammation from exercise-induced muscle damage (de Oliveira H. A. et al., 2018) and pre-conditioning the muscle before exercise, thus reducing CK concentration (Ferraresi et al., 2015; Leal-Junior et al., 2015).

The mechanisms of PBMT are complex (Zein et al., 2018) and multiple parameters need to be considered before interpreting the findings of PBMT studies. Parameters such as wavelength, fluency, irradiation, spot size, and delivery method (e.g., contact, punctual, broad beam) need to be taken into consideration. According to Zein et al. (2018), the two most important parameters to be reported are irradiance, measured in mW/cm^2^, and fluence, measured in J/cm^2^. In the current study, the irradiance and fluence of the device were 17 mW/cm^2^ and 25 J/cm^2^, respectively. At our wavelength of 660–850 nm, the irradiance value is similar to that of previous work (Wang and Wang, 2019) as well as to fluence (Zein et al., 2018). The energy emitted per LED is 164 J, the beam area per LED is 6.5 cm^2^, and the total energy emitted for the 15-min period is 473,400 J. The total energy dose in joules, however, is not in accordance with Leal-Junior et al. (2019) or Vanin et al. (2018), who recommend 60–300 J for large muscle groups and 20–60 J for small muscle groups. The large size of our total energy dose is a consequence of the use of whole-body PBMT administration. We do recognize that this dose is beyond the suggested therapeutic window and believe that it might contribute to our lack of positive findings (Vanin et al., 2018).

Although our data do not support PBMT as reducing CK concentration, our findings are similar to the conclusions seen in the review by Machado et al. (2018), that PBMT is not effective at reducing CK for general exercise compared to localized exercise. The rationale is that localized exercise provides more considerable muscle damage, thus allowing PBMT to have a greater effect on the recovery response. General exercise does not cause enough stress to see a recovery response in the sample populations, who were particularly trained and accustomed to the stress of the exercise program. Our muscle-damaging protocol was indeed not localized; however, the participants were accustomed to the basic exercises. Even with participants who were familiar with the program (i.e., bench press, chin-ups, and sprinting), a significant spike in CK from baseline to 24 hrs was evident, thus creating an environment of muscle damage for the benefits of PBMT to occur, should they exist. The CK responses were similar to that of previous work that examines the acute CK response after high-intensity resistance training (Koch et al., 2014). The muscle-damaging protocol consisted of a high volume of upper-body exercises that implemented eccentric muscle contractions, which is consistent with the literature regarding the types of protocols that elevate CK (Koch et al., 2014). Post-exercise, the typical response to CK is an elevated level after 24 h, followed by peak levels at 48 h, with a slight decrease in concentration at 72 h. Most of our participants are categorized as “high responders” based on the CK, reaching peak levels at 24 h post-high-intensity exercise (Baird et al., 2012). Several factors can account for a CK response from exercise, including age, gender, body composition, and training status (Baird et al., 2012). Secondary factors, such as training volume, selection of exercises (i.e., upper vs. lower body), and nutritional status, also can affect the response (Koch et al., 2014). We successfully controlled for diet, sleep, and nervous system recovery demonstrated by the lack of differences between PBMT and control conditions. Therefore, it is unlikely that these confounding factors impacted our null research findings.

The mechanisms that underlie muscle damage, as presented in the literature, are not universal due to inter-subject variability and the variability of the experimental training programs (e.g., intensity, volume, mode of muscle activation, muscle groups (Hyldahl and Hubal, 2014). The key factor that influences recovery from muscle damage is previous damage (Peake et al., 2016), which is known as the repeat bout effect (RBE) and is a widespread phenomenon (Hyldahl et al., 2017). RBE occurs after a bout of exercise-induced muscle damage; the muscle has an intrinsic protective mechanism that provides resistance to subsequent damage (Hyldahl et al., 2017). Our data show a 37% drop in AUC for CK from week 1 to week 2. It is possible that the RBE was present, thus attenuating the potential positive effects of PBMT. Previous authors who examine PBMT to assess recovery, control for the RBE by using contralateral limbs for the experimental treatment and placebo trials (Orssatto et al., 2019). Separating the PBMT and control treatment for different body parts was not a viable option in the current study for two reasons. PBMT bed therapy was applied to the whole body; therefore, treating individual limbs was not possible. In addition, the muscle-damaging protocol was a total-body routine to simulate a more practical approach to how this sample population would exercise. For future designs that are similar to that of the current study, we suggest implementing an extended wash-out period (i.e., 4 weeks) or the use of parallel groups.

The salivary IL-6 scores were similar to those other studies that examine saliva IL-6 in an athletic population (de Freitas et al., 2019) as well as in terms of the spike post-exercise (Minetto et al., 2005, 2007; Ives et al., 2011). Our data do not support the findings of Amadio et al. (2015) and de Oliveira H. A. et al. (2018), which showed a decrease in salivary IL-6 post-exercise from PBMT compared to the control in rat models. One factor in our study that could be related to the lack of statistical significance for salivary IL-6 is the large amount of variability within the data set. Albeit after removing one of our extreme outliers, our variability, particularly at the 60-min time point, had large standard deviations, producing an abnormal distribution that called for a non-parametric statistical analysis. In addition, in a review by Nam et al. (2019) reports salivary measures of IL-6 to have a weak correlation to blood levels. Several of these papers, however, did not control for saliva collection time or used immunoassay kits developed for saliva samples. Further, it is possible that our IL-6 saliva scores were not indicative of our participants' actual inflammatory state.

Our study had both strength and limitations. Our sample size produced an adequate power of 0.88, thus avoiding the risk of a type II error. The sample also was considered well-trained, based on the average fat-free mass index (24.5), which is indicative of Division 1 football players (Trexler et al., 2017) and Division II athletes of multiple sports (Currier et al., 2019). The training status of our population allows our findings to be generalized to those who resistance train on a regular basis. Other strengths include the fact that the PEDro scale, which is a validated assessment of the methodological quality of clinical trials (de Morton, 2009), showed that the current study has a score of 7 (“high quality”). The strengths of the randomized, counterbalanced, crossover design eliminated any preexisting individual differences between the PBMT and control groups at baseline. Limitations to be considered were that a large amount of between-subject variability existed immediate post-exercise, specifically for salivary IL-6, which caused normality violations. A second limitation (according to items 3, 5, 6, and 7 on the PEDro scale) is the lack of blinding the PBMT to the participants and primary investigator. Blinding the PBMT to the participants was not possible due to the fact that participants' knowledge of when the light beds were on or off. To the author's knowledge, a sham treatment for the full-body light bed is not currently available. A third limitation is that the whole-body bed method does allow for direct contact with the skin, which is not in accordance with the recommendations of Leal-Junior et al. (2019). This limitation is applicable to hand-held devices when the practitioner is able to place the therapeutic device directly over the target tissue. This technique, however, cannot be used with a whole-body device (specifically, on the anterior side of the body), as the top of the bed is several inches above the subject, and, thus, there is no direct contact. Nevertheless, the posterior chain is in direct contact with the bottom LEDs for the person who is lying down on the bed. A final limitation, as stated above, is the discerning presence of the RBE.

Future research should assess the efficacy of the PBMT light bed over consecutive days to better quantify its effects on recovery. Our acute dose produced a moderate effect at reducing CK concentration; however, applying the treatment consistently over a longer duration may yield statistical significance. With over a decade of research, PBMT is not new to the field of performance and rehabilitation. The methods of administration, however, are still developing, including the award-winning whole-body therapy and advancements in commercial home devices (Gavish and Houreld, 2018).

## Conclusion

The current study did not find statistical significance for the decrease in CK and salivary IL-6 concentrations through the use of whole-body bed PBMT at a dose of 15 min immediately before and after high-intensity resistance training. Thus, the findings were comparable to those of the control group. There is a growth of evidence for the use of PBMT; however, there is a lack of evidence for the use of whole-body machines.

## Data Availability Statement

All datasets generated for this study are included in the article/Supplementary Material.

## Ethics Statement

The studies involving human participants were reviewed and approved by Hofstra Univerisity: Human Research Ethics Committee 45 CFR 46.110(d). The patients/participants provided their written informed consent to participate in this study. Written informed consent was obtained from the individual(s) for the publication of any potentially identifiable images or data included in this article.

## Author Contributions

JG, AFu, and DM conceived the study design. JG, AFu, AB, AFe, JZ, and SC participated in the data collection. JG, AFu, AB, LP, AG, and KS drafted the manuscript and read and approved the final version of the manuscript. JG carried out the statistical analyses.

## Conflict of Interest

AFu was employed by the company Crux Physical Therapy. The remaining authors declare that the research was conducted in the absence of any commercial or financial relationships that could be construed as a potential conflict of interest.

## Supplementary Material

Supplementary Tables 1, 2 Reporting the CK and IL-6 values for each individual at the four time points across both conditions. It also reports the AUC and percent change calculation for each participant and the groups.

Supplementary Tables 3, 4 Reports a portion of the HRV and sleep metric data for the participants.
